# Incidence and Predictors of New-Onset Atrial Fibrillation in Septic Shock Patients in a Medical ICU: Data from 7-Day Holter ECG Monitoring

**DOI:** 10.1371/journal.pone.0127168

**Published:** 2015-05-12

**Authors:** Charles Guenancia, Christine Binquet, Gabriel Laurent, Sandrine Vinault, Rémi Bruyère, Sébastien Prin, Arnaud Pavon, Pierre-Emmanuel Charles, Jean-Pierre Quenot

**Affiliations:** 1 University Hospital, Department of Cardiology, Dijon, France; 2 INSERM, UMR866, LPPCM, Dijon, France; 3 University Hospital, Clinical Investigation Centre, Clinical epidemiology/clinical trial unit, Dijon, France; 4 INSERM, CIC1432, Clinical epidemiology unit, Dijon, France; 5 CNRS, UMR 5158, Le2I, Dijon, France; 6 University Hospital, Intensive Care Unit, Dijon, France; University of Pittsburgh, UNITED STATES

## Abstract

**Purpose:**

We investigated incidence, risk factors for new-onset atrial fibrillation (NAF), and prognostic impact during septic shock in medical Intensive Care Unit (ICU) patients.

**Methods:**

Prospective, observational study in a university hospital. Consecutive patients from 03/2011 to 05/2013 with septic shock were eligible. Exclusion criteria were age <18 years, history of AF, transfer with prior septic shock. Included patients were equipped with long-duration (7 days) Holter ECG monitoring. NAF was defined as an AF episode lasting >30 seconds. Patient characteristics, infection criteria, cardiovascular parameters, severity of illness, support therapies were recorded.

**Results:**

Among 66 patients, 29(44%) developed NAF; 10 (34%) would not have been diagnosed without Holter ECG monitoring. NAF patients were older, with more markers of heart failure (troponin and NT-pro-BNP), lower left ventricular ejection fraction (LVEF), longer QRS duration and more nonsustained supra ventricular arrhythmias (<30s) on day 1 than patients who maintained sinus rhythm. By multivariate analysis, age (OR: 1.06; p = 0.01) and LVEF<45% (OR: 13.01, p = 0.03) were associated with NAF. NAF did not predict 28 or 90 day mortality.

**Conclusions:**

NAF is common, especially in older patients, and is associated with low ejection fraction. We did not find NAF to be independently associated with higher mortality.

## Introduction

In the intensive care unit (ICU), atrial fibrillation (AF) is a common and complex phenomenon. Sepsis, and particularly septic shock, have been shown to trigger this type of arrhythmia [1, 2].

Strikingly, only one single-center prospective study has been performed to date evaluating the incidence of new-onset AF (NAF) in patients with septic shock in a non-cardiac surgical ICU. In this study, Meierhenrich et al. [3] found a 46% rate of AF among 50 patients with septic shock. However, the incidence of AF in the ICU in the context of severe sepsis and septic shock reported in previous studies [1, 3–5] should be considered with caution. In these studies, AF was diagnosed based on the ECG when cardiac monitoring showed pre-defined criteria, or AF was defined retrospectively based on the clinical diagnosis recorded in the medical files. Yet, it has been established for some time that "silent" AF can increase mortality and risk of stroke in neurology and cardiology [6–8]. Consequently, only the use of continuous long-term heart rate monitoring could firmly establish the real incidence of NAF and its characteristics.

The identified predictors of AF in sepsis include advanced age, renal failure, preexisting cardiopathy, history of hypertension, high SOFA score, high CRP levels [3] and previous calcium-channel blocker treatment [1]. However, no study to date has examined the electrocardiographic or echocardiographic features of AF, such as P-wave duration or left atrial area, although both these factors are well known in the field of cardiology to predict occurrence of AF [9].

Almost 15% of patients in the ICU suffer from sepsis (severe sepsis and septic shock), and mortality rates remain high (30 to 48% at 28 days), despite an improvement in mortality rates over the last decade [10, 11], with even more pronounced declines outside of Europe [12, 13]. In the context of sepsis, Walkey et al. [14] recently described an increased risk of in-hospital mortality and stroke in patients with NAF. However, it remains unclear whether any type of AF (silent or symptomatic AF) impairs prognosis, regardless of its duration or its ventricular response.

The main purpose of this study was to determine, using 7-day Holter ECG monitoring, the incidence of NAF in patients with septic shock hospitalized in a non-surgical ICU. Secondary objectives were to perform a preliminary exploration of the predictive factors and of the characteristics of NAF (silent or symptomatic).

## Methods

This study was performed in a medical 15-bed ICU between March 2011 and May 2013. This unit only admits non-surgical patients requiring hemodynamic support, respiratory, circulatory or renal assistance or special observation. The study was approved by the local ethics committee (Comité de Protection des Personnes (CPP) Est I, Dijon, France). The study was approved by the local ethics committee (Comité de Protection des Personnes (CPP) Est I, Dijon, France). The study was considered to be part of routine care and the need for written informed consent was waived by the local ethics committee. All patients or their proxies were informed about the study.

### Study design

This was a prospective, single-center, observational study. During the study period, all patients aged >18 years with a confirmed diagnosis of septic shock in the ICU were included.

Exclusion criteria were patients with known AF (paroxysmal or sustained), and patients transferred from another ICU with prior septic shock. This information was obtained at admission from the patient’s general practitioner or cardiologist, during verification of inclusion/exclusion criteria. We did not exclude patients with other known arrhythmias, or patients with implantable cardiac devices.

Two groups of patients were created after Holter ECG monitoring analysis: NAF patients and patients who maintained sinus rhythm.

Clinical data collection was exhaustive at admission and the following variables were recorded: sex, age, cardiovascular risk factors, cardiovascular, thyroid and pulmonary diseases, outpatient medications and ECG analysis (P wave duration, QRS duration, QT length with Bazett correction).

In the first 24 hours after admission, transthoracic echocardiography was performed and total fluid balance was calculated (crystalloids and colloids). During the stay in the ICU, all medications administered (but not the doses) were recorded. Blood samples were taken at admission and then every 24 hours to assess the following variables: total blood count, electrolytes, corrected calcemia, creatinine, cardiac troponin I, NT pro-BNP, procalcitonin, lactates, blood protein levels (delta total protein level calculation: percentage variation of total protein level during ICU stay between maximum and minimum value), TSH, T3, T4. Simplified Acute Physiological Score (SAPS) II (17) was recorded at admission to the ICU, and Sequential Organ-related Failure Assessment (SOFA) score (18) over the first 24 hours following vasopressor initiation was also recorded.

Lastly, we recorded clinical outcomes over 90 days follow-up after discharge from the ICU, namely: length of stay in the ICU and in the hospital, mortality in the ICU, at discharge (or day 28, whichever came first) and at 90 days. All patients were followed-up, and follow-up information at 90 days was obtained by telephone contact either with the patient, the patient’s family or the general practitioner.

### Definition of septic shock

Septic shock was defined as in previous studies [11, 15] namely documented or suspected infection requiring initiation of vasopressors despite adequate vascular filling, with at least one of the following hypoperfusion criteria: (i) metabolic acidosis (base excess ≥5 mEq/L, alkaline reserve <18mEq/L or lactate ≥ 2.5 mmol/L); (ii) oliguria/renal insufficiency (<0.5 ml/kg/h for 3h or elevation >50% of baseline creatinine); or (iii) hepatic dysfunction (AST or ALT >500 IU/L or bilirubin >34 μmol/L).

#### Diagnosis of new-onset atrial fibrillation

All patients underwent Holter ECG monitoring (Spider Flash, Sorin Group France) started immediately after inclusion. The Holter ECG device was programmed to record every rhythmic event for 7 days, regardless of the duration of the supraventricular arrhythmia. The device was consistent with routine use in the ICU since only 3 electrodes were required, thus providing a 2 lead recording, and was easily removable if necessary for patient care or to perform imaging exams. The Holter ECG monitor was removed after 7 days of recording, or at discharge/death (whichever occurred first). An experienced cardiologist who was blinded to the patient’s clinical data performed the Holter ECG analysis. If the diagnosis was uncertain, a second cardiologist, blinded to the first results, also analyzed the records. No case of discordance between both analyses occurred.

The diagnosis of AF was made according to the guidelines of the European Society of Cardiology (ESC) for the interpretation of Holter ECG recordings, i.e. any arrhythmia that presents the ECG characteristics of AF (namely, “the surface ECG shows ‘absolutely’ irregular RR intervals, there are no distinct P waves on the surface ECG, and the atrial cycle length (when visible), i.e. the interval between two atrial activations, is usually variable and <200 ms (>300 bpm)”) and lasting at least 30s on a rhythm strip, should be considered as AF [9]. Silent AF was defined as the occurrence of AF on the Holter ECG monitoring, regardless of the duration or number of episodes, and in the absence of any mention of AF in the medical file during the ICU stay (i.e. AF non diagnosed by the ICU physicians) [7].

When AF was diagnosed on the Holter ECG recording, the following data were collected: date and time of the first episode, ventricular response to the first episode and mean ventricular response in AF, total burden of AF (total duration of all episodes of AF), number of episodes. The number of premature atrial and ventricular contractions and non-sustained supraventricular tachycardias (<30s) was also recorded from the automated count for all patients, and the daily mean ventricular frequency was calculated. In patients requiring prone positioning, the Holter ECG electrodes were stuck to the patient’s back.

### Treatment of new-onset AF

After discharge, each patient’s daily medical files and treatment sheets were analyzed by a physician unaware of the Holter ECG monitoring results, to evaluate the number of AF cases diagnosed by the clinicians (symptomatic AF) and the treatment choices (rate control, medical cardioversion, anticoagulant therapy).

### Echocardiography

Transthoracic echocardiography and Doppler examination were performed in all patients by experienced operators using HD11XE (Philips Healthcare, Andover, MA, USA). Measurements were taken in triplicate at end-expiration. LVEF was obtained using the biplane method (modified Simpson's rule) in the apical two-chamber and four-chamber views. Systolic dysfunction was defined as LVEF <45%. Tricuspid annular plane systolic excursion (TAPSE) was obtained in TM mode at the lateral tricuspid annulus. Transmitral velocities were recorded with pulsed-wave Doppler with the sample volume placed at the mitral valve tips in the apical four-chamber view. Peak passive (E) and active (A) velocities were recorded. E to A ratio was calculated. Myocardial velocities were obtained using tissue Doppler settings, with the pulsed-wave Doppler sample volume at the septal and lateral mitral annulus and at the lateral tricuspid annulus in the apical four-chamber view. Peak early diastolic (e') and late diastolic (a') myocardial velocities were measured. E/e' was calculated. Peak systolic (S't) myocardial velocities at the tricuspid annulus were measured. In the presence of atrial arrhythmia, transmitral and tissue Doppler velocities were measured over five consecutive cycles. As described by Sturgess et al. [16], the threshold for abnormal diastolic tissue Doppler imaging was defined as e' <9.6 cm/s or E/e' >15. Right ventricular dysfunction was defined as S wave < 11 cm/s or TAPSE <17 cm and classified as moderate (S between 9 and 11 cm/s, or TAPSE between 13 and 17 cm) or severe (S<9 cm/s or TAPSE<13 cm)[17].

### Statistical analysis

The study was designed to estimate incidence with a precision of 5% (corresponding to a confidence interval of ±5 around the true value), at an alpha risk of 0.05 and a beta risk of 0.10) based on a hypothetical incidence of 10 per 100 patient-days.

Continuous variables are presented as mean± standard deviation (SD) when normally distributed or median and range otherwise; and categorical variables as number (percentage). The characteristics of the AF and sinus rhythm groups were compared using the exact Mann-Whitney test for continuous variables and Fisher's exact test for categorical variables. Exploratory identification of factors associated with AF was performed using logistic regression. The two baseline variables significantly related to AF by bivariate analysis (p<0.05) were included in the model (age and LVEF<45%). Characteristics associated with silent AF versus other AF were investigated using the same strategy.

For all analyses, a *P*-value <0.05 was considered statistically significant. All analyses were performed using SAS version 9.3 (SAS Institute Inc., Cary, NC, USA).

## Results

### Study population

Between March 2011 and May 2013, a total of 1071 patients were admitted to the participating ICU and prospectively screened. Among these, 150 (14%) presented septic shock, of whom 76 (51%) met the inclusion criteria. Among the 74 patients excluded, 36 had previous AF, and 38 were addressed from another ICU with previous septic shock. Ten of the 76 potentially eligible patients were not included in the study (4 because no Holter ECG monitor was available at inclusion and 6 because of technical problems (missing Holter ECG records)). However, there was no significant difference in baseline values between the 66 patients included in the study and the 10 patients with missing data. Thus, 66/150 septic shock patients (44%) were included in the study.

### Incidence of new-onset AF in septic shock

During the study period, AF occurred in 29/66 patients (44%); the remaining 37(56%) remained in sinus rhythm.

**Fig 1:**
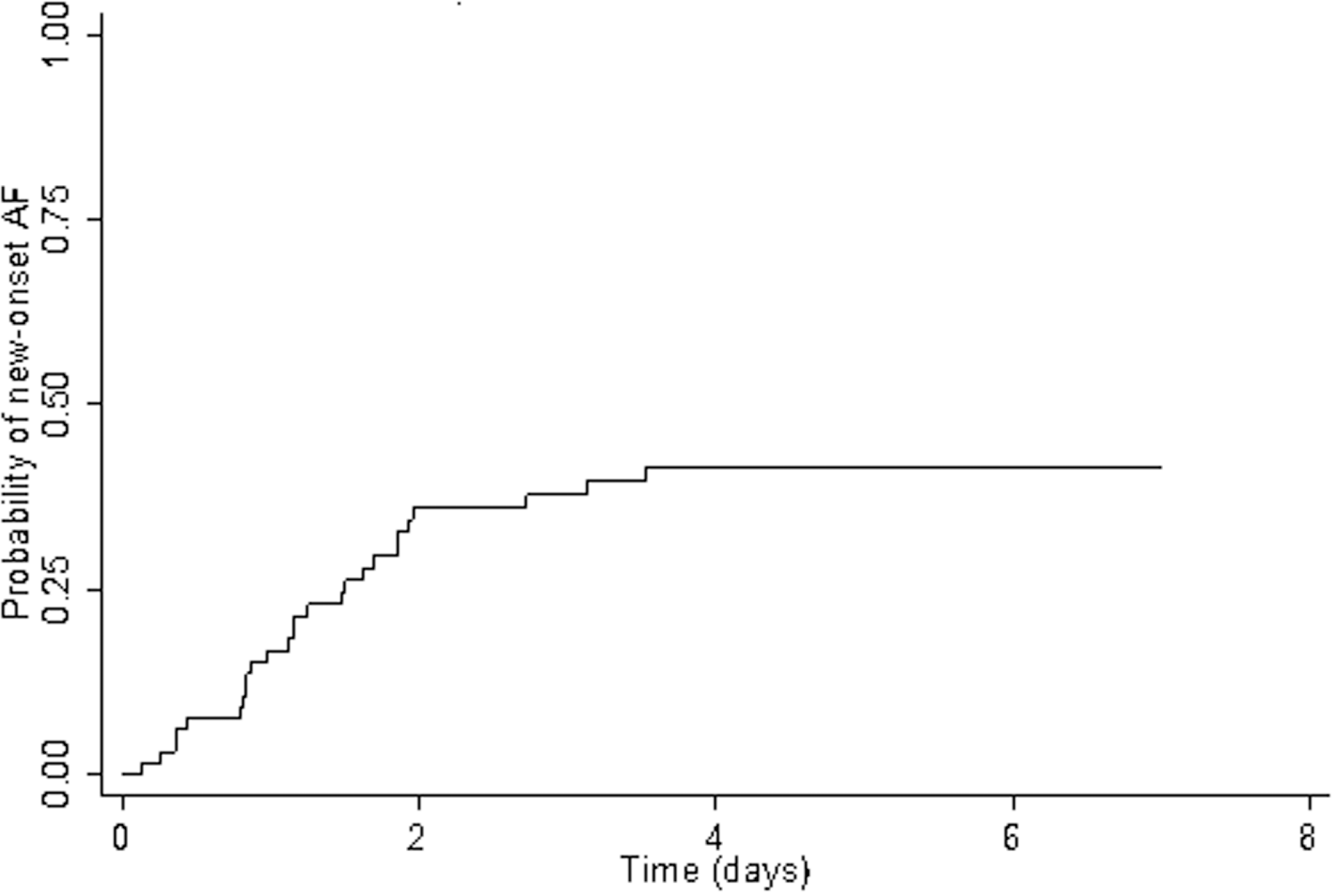
Probability of new-onset AF during the 7 first days of septic shock (Kaplan-Meier curve).

The incidence of first episodes of AF during the 7 first days of septic shock was 12.0 (8.3–17.2) AF per 100 patient-days. Fig 1 illustrates the probability of AF occurrence.

The comparisons between septic shock patients with new-onset AF vs those who remained in sinus rhythm are reported in Tables 1–4. In terms of baseline characteristics (Table 1), septic shock patients with NAF were significantly older than patients who maintained sinus rhythm (p< 0.001), but there was no significant difference in terms of other cardiovascular risk factors or previous cardiac or non-cardiac diseases. Moreover, previous medications were similar in both groups. Similarly, there was no significant difference in severity of illness (SAPS II or SOFA scores), or septic shock characteristics between groups (Table 2).

**Table 1:** Baseline Characteristics According To The Onset Of Atrial Fibrillation.

n (%), median [interquartile range], mean ± SD	New-onset AF	Sinus rhythm	P
	n = 29 (44%)	n = 37 (56%)	
*Risk factors*			
Age, years	71.0 ±13.5	60.5 +/- 13.5	<0.001
Female sex	11 (37.9)	13 (35.1)	0.81
Systemic hypertension	16 (55.2)	18 (48.7)	0.60
Hypercholesterolemia	11 (37.9)	7 (18.9)	0.08
Active Smoking	4 (13.8)	12 (32.4)	0.08
Diabetes mellitus	7 (24.1)	7 (18.9)	0.61
BMI, m^2^/kg	26.3 [24.6–29.3]	24.6 [21.1–30.1]	0.17
*Outpatient medications*			
B-blocker	9 (31.0)	8 (21.6)	0.39
Calcium antagonist	9 (31.0)	5 (13.5)	0.08
ACE inhibitor	11 (37.9)	8 (21.6)	0.15
Diuretic	6 (20.7)	7 (18.9)	0.86
Antiplatelet therapy	7 (24.1)	4 (10.8)	0.19
VKA	4 (13.8)	1 (2.7)	0.16
*Medical history*			
CAD	6 (20.7)	4 (10.8)	0.31
Valvular disease	1 (3.5)	2 (5.4)	1
Other cardiopathy	10 (34.5)	8 (21.6)	0.24
Previous hyperthyroidism	2 (6.9)	1 (2.7)	0.58
Previous hypothyroidism	5 (17.2)	2 (5.4)	0.23
Previous pulmonary embolism	1 (3.5)	1 (2.7)	1
Immunodepression	14 (48.3)	12 (32.4)	0.19
Previous health status (Knaus)			0.65
A	3 (10.3)	6 (16.2)	
B	18 (62.1)	19 (51.4)	
C	8 (27.6)	10 (27.0)	
D	0	2 (5.4)	

ACE indicates angiotensin conversion enzyme; AF: atrial fibrillation; BMI: body mass index; CAD: Coronary Artery Disease; SD: standard deviation; SR: Sinus Rhythm; VKA: Vitamin K Antagonists.

**Table 2:** Clinical and Biological Characteristics According To The Onset Of Atrial Fibrillation.

n (%), median [interquartile range], mean ± SD	New-onset AF	Maintained SR	P
	n = 29 (43.9)	n = 37 (56.1)	
*Severity of illness scores*			
SAPS II score	56 [45–71]	50 [39–57]	0.13
SOFA score	10.9 +/- 2.9	10.9 +/- 3.2]	0.89
*Septic shock characteristics*			
Metabolic acidosis	26 (89.7)	32 (86.5)	1
Oliguria/ Renal failure	18 (62.1)	23 (62.2)	0.99
Hepatic dysfunction	4 (13.8)	10 (27.0)	0.19
Surgical Emergency	2 (6.9)	1 (2.7)	0.58
Nosocomial infection	6 (20.7)	10 (27.0)	0.55
*Site of infection*			
Pulmonary	19 (65.5)	26 (70.3)	0.68
Urinary	7 (24.1)	6 (16.2)	0.42
Gastrointestinal	5 (17.2)	6 (16.2)	1
*7 first days biological data*			
Anemia <10g/dl	23 (79.3)	30 (81.1)	0.86
Hyperthyroidism	6 (20.7)	8 (26.7)	0.59
Hypercalcemia	12 (41.4)	10 (27.0)	0.22
Hyperkaliemia	7 (24.1)	6 (16.2)	0.42
Hypokaliemia	22 (75.9)	32 (86.5)	0.27
Lactate peak, mmol/l	6.5 +/- 6.2	6.3 +/- 7.0	0.72
Troponin peak>100 ULN	9 (32.1)	2 (5.6)	<0.01
NT pro BNP peak, pg/ml	9433 [4988–27745]	2536 [798–9490]	0.01
Procalcitonin peak, μg/L	30.4 [3.5–153.0]	13.7 [2.6–32.1]	0.09
Delta total protein level (max-min)/max, g/l	0.24 [0.17–0.29]	0.20 [0.15–0.32]	0.82

AF indicates atrial fibrillation; NT pro BNP: n terminal pro brain natriuretic peptide; SAPS II: simplified acute physiological score; SOFA: sequential organ failure assessment

**Table 3:** Rhythmic Characteristics According To The Onset Of Atrial Fibrillation.

n (%), median [interquartile range], mean ± SD	New-onset AF	Maintained SR	P
	n = 29 (43.9)	n = 37 (56.1)	
*ECG at admission*			
P wave duration >100ms	6 (25.0)	3 (9.1)	0.15
QRS duration (ms)	86.5 [80.0–100.0]	80.0 [80.0–90.0]	0.04
QRS duration >120 ms	5 (19.2)	1 (3.0)	0.08
QTc duration (ms)	431.5 [414–467]	412.0 [382–454]	0.07
*Holter ECG monitoring*			
PAC day 1	110 [73–131]	87 [66–110]	0.11
PVC day 1	60 [36–93]	48 [32–76]	0.56
NSVT day 1	2.5 +/- 7.0	0 +/- 0	<0.01
Time delay between admission and first AF record (days)	1.7 +/- 1.5		
Total duration of AF during Holter ECG monitoring (hours)	18.6 +/- 35.4		
Number of AF episodes during Holter ECG monitoring	1.8 +/- 1.4		
Silent AF	10 (34.5)		

AF indicates atrial fibrillation; ECG: electrocardiogram; QTc: corrected QT; NSVT: Nonsustained Supra ventricular tachycardia; PAC: premature atrial contraction; PVC: premature ventricular contraction.

**Table 4:** Echocardiographic Characteristics According To The Onset Of Atrial Fibrillation.

n (%), median [interquartile range], mean ± SD	New-onset AF	Maintained SR	P
	n = 29 (43.9)	n = 37 (56.1)	
LVEF <45%	7 (25)	1 (3)	<0.01
LVEF	51 +/- 13	59 +/- 6	<0.01
*Mitral Regurgitation*			
Mild	8 (27.6)	9 (25.7)	0.87
Moderate or severe	2 (6.9)	2 (5.7)	1
Pericardial effusion	2 (6.9)	2 (5.4)	1
Left atrial surface >20 cm^2^	10 (55.6)	10 (41.7)	0.37
*Diastolic function*			
E velocity	0.75 [0.66–0.95]	0.80 [0.59–1.03]	0.95
E/e’	9.9 [6.0–10.7]	8.0 [6.3–10.0]	0.46
Diastolic dysfunction	13 (54.2)	10(41.7)	0.39
TAPSE	22.4 [16.0–23.6]	22.6 [19.0–25.0]	0.27
S Wave	16.6 [13.0–18.3]	15.2 [12.3–17.3]	0.69
Right ventricular dysfunction	7 (36.8)	3 (11.5)	0.07
Elevated Right Atrial Pressure (IVC>2cm)	6 (35.3)	5 (20.0)	0.30

AF indicates atrial fibrillation; IVC: inferior vena cava; LVEF: left ventricular ejection fraction; TAPSE: tricuspid annular plane systolic excursion.

### Factors associated with new-onset AF

Regarding the initial gravity of septic shock, no significant difference was observed between groups in terms of SAPS II or SOFA scores. Similarly, average lactates peak was similar in both groups, as were the characteristics of sepsis.

Cardiac parameters were markedly different in AF patients compared to patients in sinus rhythm, both in terms of electrocardiographic and echocardiographic measurements (Tables 3 and 4): they had significantly longer QRS duration at admission ECG (86.5 vs 80.0, p = 0.04), more frequently had impaired LVEF (<45%) (p<0.01), higher cardiac troponin I and NT pro-BNP peaks (9±32 vs 2±6, p<0.01 for troponin peak; 9433 vs 2536, p = 0.01 for NT pro BNP). Moreover, the Holter records on the first day showed a higher rate of nonsustained supra ventricular tachycardia (p<0.01) in AF patients.

Biological examinations performed during ICU stay did not show serum electrolyte levels, anemia or thyroid disturbance to be associated with the development of AF.

By multivariate logistic regression, only age (OR: 1.06; 95% CI: 1.01–1.11, p = 0.01) and impaired LVEF <45% (OR: 13.01; 95% CI: 1.36–124.19, p = 0.03) were shown to be independently associated with NAF.

### Silent AF

Approximately one third of AF (10/29) were defined as silent AF, since they were not diagnosed by clinicians during the ICU stay. Compared with symptomatic AF patients, patients with silent AF less often had immunodepression (20% vs 63%, p = 0.05), while all others parameters (baseline characteristics and outcomes) were not significantly different.

### Outcomes

ICU mortality rate in septic shock patients with and without new-onset AF was comparable (24% vs 19%, p = 0.61). Moreover, the duration of vasopressive drugs, mechanical ventilation and the use of hemodialysis were comparable between AF patients and those in sinus rhythm (Table 5).

**Table 5:** In-ICU Management According To The Onset Of Atrial Fibrillation.

n (%), median [interquartile range], mean ± SD	New-onset AF	Maintained SR	P
	n = 29 (43.9)	n = 37 (56.1)	
*Treatment*			
Norepinephrine duration (days)	4 [3–7]	3 [2–5]	0.11
Mechanical Ventilation	22 (75.9)	32 (86.5)	0.27
Mechanical Ventilation duration (days)	10 [7–16]	8 [5–16]	0.50
*Hemodialysis*	16 (55.2)	14 (37.8)	0.16
CVVHD duration (days)	5 [3–6]	6 [5–9]	0.55
Intermittent hemodialysis (number)	4 [2–6]	3 [2–3]	0.78
Hydrocortisone	19 (65.5)	26 (70.3)	0.68
Inotropic catecholamine	4 (13.8)	2 (5.4)	0.39
Anticoagulation	22 (75.9)	10 (27.0)	<0.01
Amiodarone	19 (65.5)	3 (8.1)	<0.01
*First 24 hours fluid balance*			
Crystalloids (l)	2.9 +/- 1.5	3.0 +/- 1.9	0.98
Colloids (l)	0.3 +/- 0.5	0.3 +/- 0.5	0.52
*Follow up*			
ICU stay duration (days)	10 [4–17]	7 [4–14]	0.76
ICU death	7 (24.1)	7 (18.9)	0.61
In-hospital stay duration (days)	22 [14–41]	26 [8–40]	1
In-hospital death	9 (31.0)	9 (24.3)	0.54
90 days death	12 (41.4)	16 (43.2)	0.88

AF indicates atrial fibrillation; CVVHD: continuous veno-venous hemodialysis; ICU: intensive care unit.

Mortality did not differ significantly between groups at 28 days (22% vs 28%, NAF vs sinus rhythm respectively, p = 0.58) or at 90 days (41% vs 43% respectively, p = 0.88). Among survivors at 90 days follow-up, patients with septic shock and new-onset AF had a similar duration of stay in the ICU (median stay 9 days), to those with septic shock and sinus rhythm (median stay 7 days, p = 0.77). No significant difference was found in ICU management between the 2 groups.

## Discussion

The main finding of this prospective observational study is the real-life incidence of NAF in patients hospitalized in a medical ICU with a septic shock (12.0 per 100 patient-days). In our study, the use of continuous Holter ECG recording during the first 7 days of the ICU stay showed for the first time not only the incidence, but also the features of NAF.

The frequency of 44% described in the present study is very close to the results reported by Meierhenrich et al [3] from the first prospective study of AF in septic shock, performed in 50 patients in a non-cardiac surgical ICU. These authors observed NAF in 46% of the population, despite only using continuous monitoring for AF screening. In our study, this strategy would have led to an observed frequency of 28%. In fact, 10 of the 29 AF observed in our study were not diagnosed during the ICU stay (so-called silent AF), but only afterwards, on the basis of the 7-day Holter recordings. This frequency is closer to that reported in the study by Seguin et al [1], in which 7 of a subgroup of 23 (30%) septic shock patients in a surgical ICU developed AF (diagnosed using the same method as in Meierhenrich’s study [3]). The high incidence of NAF in our study could also be explained by the septic shock definition we used. In fact, we probably recruited more severe patients than if we had used the Consensus Statement definition of 2001[18] In a retrospective, population-based cohort, Walkey et al [14] observed an incidence of 5.9% NAF in 49,082 severe sepsis patients, by collecting the diagnostic codes of both septic shock and AF. In a second study among 26,412 Medicare beneficiaries, the same authors found that 10.7% of patients hospitalized for sepsis requiring ICU stay developed NAF [19]. The wide range of AF frequencies underlines the fact that the diagnosis of AF is closely dependent on the definition and the method of screening. The main interest of our study is that, using continuous 7 day Holter monitoring, we were able to precisely analyze on a 2 lead record any arrhythmia meeting the ESC definition of AF on a Holter recording, thus providing the actual incidence of AF, both silent and symptomatic. To the best of our knowledge, this is the first time that such a device is used in the ICU context for AF screening.

Among the baseline characteristics, only age was a significantly associated with AF in septic shock. Advanced age is a strong risk factor for AF in the general population, but also in ICU patients [1, 20] and particularly septic shock [3]. Unlike previously mentioned studies, AF was not found to be associated with greater shock severity in our study. However, unlike Seguin et al [1], we did not record SOFA score daily during the ICU stay. Nonetheless, the data on catecholamines, mechanical ventilation and hemodialysis use and duration, combined with lactates peak and mortality made it possible to assess the severity of shock.

We sought to determine whether the occurrence of AF during septic shock could be linked to baseline and dynamic cardiac parameters. In our study, every patient underwent 12-lead ECG at admission, and we observed that QRS duration was significantly higher in AF patients. This result cannot be linked to a higher prevalence of previous cardiac disease, as the rate of previous cardiopathy was similar in both groups. QRS duration has been shown to be strongly associated with AF in patients with LV dysfunction after adjusting for disease severity, comorbid conditions, and the use of medications known to protect against AF [21]. Thus, this increased QRS duration could be related to acute heart failure, as assessed by the higher prevalence of impaired LVEF and the higher levels of NT pro-BNP and cardiac troponin I in AF patients. The increased QRS duration could also be a marker of cardiac fibrosis, which is a known substrate of AF [22]. In fact, QRS duration slightly increases with age in patients and is an independent predictor of cardiovascular mortality in a general medical population [23]. Another novel finding to emerge from the continuous Holter monitor was the fact that occurrence of AF was associated with a state of “excitability” of the myocardium. This was reflected by the higher number of non-sustained supra ventricular tachycardia at day 1 and the trend towards a higher number of PAC on the first day.

Interestingly, the classic AF risk factors in the general population, such as hypertension, P wave duration, left atrial area, or previous cardiovascular diseases did not appear to be predisposing factors to AF in our population of septic shock patients. This finding is consistent with the recent study by Walkey et al among Medicare beneficiaries hospitalized for sepsis [19], in which acute factors, rather than preexisting cardiovascular comorbid conditions, were associated with increased risk of newly diagnosed AF during sepsis.

Global analysis of these results suggests that symptomatic AF in septic shock cannot be considered as being similar to “medical” AF occurring outside the context of septic shock, and may reflect an acute state of myocardial injury. Using an unprecedented range of cardiac examinations, the present study identified a strong association between onset of new AF and markers of heart failure (LVEF, troponin, NT-pro BNP). These markers are known to be associated with the septic myocardial dysfunction, with a poor short-term prognosis [24].

Finally, contrary to the results of previous studies, we found no significant difference in terms of outcomes. The large cohort study by Walkey et al reported that symptomatic AF in severe sepsis was associated with an increased in-hospital risk of stroke and death [19]. Our results could be explained by the relatively small sample size, but Meierhenrich et al [3] observed that NAF increased ICU stay in surviving patients (34/50) with septic shock. However, the inclusion criteria defining septic shock were more restrictive than ours, as they required a high volume and longer duration of catecholamine infusion. Thus, the patients included may have been more severe than in our study, thus developing more outcomes. Furthermore, the present study included patients in a medical ICU, and the underlying pathologies involved may differ from a surgical ICU.

No arterial embolic event occurred in the patients 90 days follow up, thus arguing for a relative low risk of embolism. However, retrospective studies on 49,082 patients with severe sepsis based on diagnosis codes found a higher incidence of stroke and mortality in case of new-onset AF [14].

### Study Limitations

Our study suffers from several limitations that deserve to be acknowledged. The major limitation is the small sample size, despite 2 years of recruitment in a single center. In practice, many patients admitted for septic shock had had previous AF (almost 23%) and septic shock remains a relatively rare pathology (14% of all patients admitted to our ICU). Moreover, 10 patients among the 76 potentially eligible (13%) had to be excluded because Holter ECG records were not available; however, these patients did not significantly differ from the 66 patients who were included in terms of baseline data. A total of 66 patients with septic shock were thus prospectively included and analyzed, which is 16 more than the only prospective study to date [3]. However, our population size did not make it possible to precisely describe the associated risk factors and to examine the impact of AF characteristics on the prognostic value of NAF during septic shock. Another limitation is the fact that severity scores were not calculated on a daily basis. However, data on the presence and duration of hemodynamic, renal and respiratory assistance, combined with the peak lactates can be considered as good surrogates for illness severity. Finally, the lack of prognostic value of the onset of NAF could be related to an “immortal time bias” whereby patients must survive long enough to get AF, and thus have a 'head start' on better survival than those without AF [25]. However, the time delay from admission to death did not differ significantly between NAF and SR patients.

## Conclusions

The incidence of NAF, especially silent AF, is particularly high in patients with septic shock. Further studies in larger septic shock populations are required to evaluate its prognostic impact in these patients. From a therapeutic point of view, it remains to be shown whether AF characteristics could guide therapeutic interventions, particularly regarding the decision for acute and chronic anticoagulation.
